# Ultra-diluted *Folliculinum 6 cH* impairs ovine oocyte viability and maturation after *in vitro* culture

**DOI:** 10.1590/1984-3143-AR2019-0100

**Published:** 2020-06-18

**Authors:** Kayse Najara Matos Damasceno, Naiza Arcângela Ribeiro de Sá, Gildas Mbemya Tetaping, Victor Macedo Paes, Laritza Ferreira de Lima, Antônio Carlos Duenhas Monreal, Francisca Geovânia Canafistula de Sousa, Bênner Geraldo Alves, José Ricardo de Figueiredo, Valdevane Rocha Araújo

**Affiliations:** 1 Laboratório de Manipulação de Oócitos e Folículos Pré-Antrais, Faculdade de Veterinária, Universidade Estadual do Ceará, Fortaleza, CE, Brasil; 2 Laboratório de Biotecnologia para Pequenos Ruminantes, Faculdade de Ciências Farmacêuticas, Alimentação e Nutrição, Universidade Federal de Mato Grosso do Sul, Campo Grande, MS, Brasil; 3 Laboratório de Biologia da Reprodução, Instituto de Ciências Biomédicas, Universidade Federal de Uberlândia, Uberlândia, MG, Brasil; 4 Ciência do Centro de Saúde, Universidade Estadual do Ceará, Fortaleza, CE, Brasil

**Keywords:** culmulus-oocyte complex, homeopathy, *in vitro* maturation, sheep

## Abstract

This study investigated the effect of *Folliculinum 6 cH* on the oocyte meiosis resumption and viability rates, progesterone production and mitochondrial activity after *in vitro* maturation of cumulus-oocyte complexes (COCs) in sheep. Sheep ovaries were collected at a local slaughterhouse and COCs were recovered by slicing technique. The selected COCs were maturated in TCM199 (Control treatment), or control medium supplemented with 0.05% ethanol (v/v) (the vehicle of the homeopathic preparation – Ethanol treatment) or with *Folliculinum 6 cH*. After 24 h of *in vitro* maturation (IVM), oocytes were mechanically denuded and incubated with Hoechst 33342 and MitoTracker (0.5 μM) Orange CMTMRos for analysis of viability and chromatin configuration, and mitochondrial activity, respectively. The results showed that *Folliculinum 6 cH* addition increased oocyte degeneration and reduced meiotic resumption compared to the control (P < 0.05). Interestingly, the percentages meiotic resumption and oocyte maturation were lower in the *Folliculinum 6 cH* treatment compared to its vehicle (Ethanol treatment) (P < 0.05). On the other hand, when the treatments were compared, higher mitochondrial activity was observed in the Ethanol treatment (P < 0.05). In conclusion, contrary to its vehicle, the addition of *Folliculinum 6 cH* to the IVM medium promoted oocyte degeneration and affected negatively the mitochondrial distribution, impairing meiosis resumption.

## Introduction

Homeopathy is a therapeutic method based on the use of drugs in a minimal dosage during treatment (Teixeira et al., 2008). In human reproduction, homeopathic preparations have been used in women who are intolerant to exogenous estrogen (Goswani and Conway, 2005) to treat various reproductive disorders, such as hyperandrogenism, dysmenorrhea, endometriosis, uterine fibroids (Legros, 2010), among others. Among the homeopathic preparations it is possible to highlight *Folliculinum 6 cH*, which is a homeopathic medicine derived from estrone (Mareüil, 2016) and has been used in women to regulate the menstrual cycle, to treat reproductive diseases, such as polycystic ovary, secondary amenorrhea after childbirth (Legros, 2010), and breast cysts in patients with the hyperestrogenic syndrome (Pordes and Legru-Bertagne, 2012) as well as for infertility treatment (Legros, 2010). Although the mechanism of action of Folliculinum 6 cH is unknown, some studies suggested that *Folliculinum 6 cH* can regulate the production of estrogens and androgens (Legros, 2010; Danno et al., 2013). Shirazi and Moalemian (2007) reported that the sheep COCs and cumulus cells are capable to produce estradiol in detectable amounts in a steroid-free maturation medium. Also, it is common to add commercial estradiol or serum containing estradiol to the culture medium to improve *in vitro* maturation (Brevini et al., 2005; Fang et al., 2016; Wang et al., 2018). Therefore, the use of *Folliculinum 6 cH* in *in vitro* procedures, such as *in vitro* maturation (IVM), could be an alternative to improve oocyte maturation in many species. Moreover, the advantages of using homeopathic substances including Folliculinum 6 cH are due to the much lower cost and low toxicity of homeopathic medicines.

Despite been used for more than 200 hundred years, homeopathy is still a controversial topic. For instance, it has been suggested that researchers have not been able to develop objective measures that show the effects of extremely dilute products in the human body. Others argued that the supposed effect of homeopathic products is due to their vehicles, such as ethanol or even to a possible placebo effect (Moffett et al., 2006). In this sense, the *in vitro* models, for example the IVM, could represent an excellent tool to solve such intriguing issues. Concerning reproduction, few studies investigated the effects of homeopathic medicinal products on the *in vitro* preantral follicle survival and development (sheep – Lima et al., 2016 and pig - Lima et al., 2017). However, to the best of our knowledge, there are no reports on the *in vitro* effects of those products on the *in vitro* maturation of oocytes. Therefore, the originality of the present paper is to investigate for the first time the effect of *Folliculinum 6 cH* and its vehicle (ethanol) on the *in vitro* maturation, viability and mitochondrial activity of ovine oocytes.

## Materials and methods

Unless mentioned otherwise, the reagents and chemicals used in the present study were purchased from Sigma Chemical Co. (St. Louis. MO. USA). The preparation of *Folliculinum* 6 cH was performed in Pharmacy Homeovitae (Campo Grande, MS).

### Research ethics

One of the major alternatives to in vivo animal testing is *in vitro* cell culture. In line with this ethical issue, the present study aimed to evaluate the effects of the tested substances (ethanol and *Folliculinum 6 cH* preparations) on *in vitro* folliculogenesis using sheep follicles recovered from slaughterhouse ovaries. This source of ovarian material represents a by-product of the food industry and is more readily acceptable than euthanasia of animals specifically for scientific purposes.

### Oocyte collection and *in vitro* maturation (IVM)

**Figure 1:**
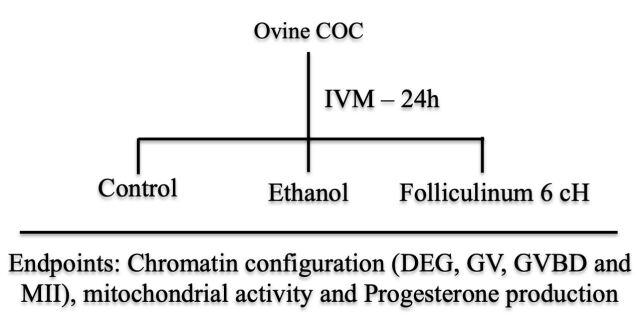
Experimental design to assess the effect *Folliculinum 6 cH* on the oocyte chromatin configuration, mitochondrial activity and progesterone production after 24 h of IVM. Control treatment- control medium alone; Ethanol - control medium supplemented with 0.05% ethanol (v/v) (the vehicle of the homeopathic preparation- Ethanol treatment); *Folliculinum 6 cH* - control medium supplemented with *Folliculinum 6 cH*.

Ovaries were collected at a local slaughterhouse and transported within 1 to 2 h to the laboratory in Minimum Essential Medium (MEM) supplemented with HEPES and antibiotics (100 µg/mL penicillin-streptomycin) at 33 to 35 °C. In the laboratory, the COCs were recovered from sheep ovary by slicing and only oocytes with homogeneous cytoplasm and surrounded by at least three compact layers of cumulus cells were selected and 20 to 35 oocytes were cultured together (Davashi et al., 2014). The basic *in vitro* maturation medium consisted of TCM199 plus sodium bicarbonate (TCM 199 B - supplemented with 0.5 µg/mL of recombinant bovine FSH (Nanocore, Brazil), 5 µg/mL of LH, 1 µg/mL of 17 β-estradiol, 10 ng/mL of murine EGF (Sigma - E4127), 0.911 mM/L of pyruvate, 100 µM/L of cysteamine, 50 ng/mL of recombinant human IGF-1 (Sigma - I3769), and 1% of BSA - Luz et al., 2013) which was referred to as TCM 199 (Control medium). Groups of 20 to 35 oocytes were cultured for 24 hours under 5% CO_2_ in air in 200-350 μL (10 µL per COC) in TCM199 (Control treatment), or control medium supplemented with 0.05% ethanol (v/v) (the vehicle of the homeopathic preparation- Ethanol treatment) or with *Folliculinum 6 cH* (Figure 1).

### Viability, chromatin configuration, and mitochondrial activity in *in vitro* matured oocytes

**Figure 2:**
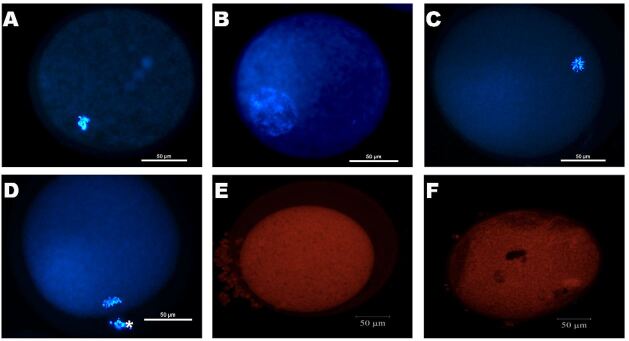
Representative images of fluorescent ovine oocytes after 24 hours of *in vitro* maturation. Oocytes staining with Hoescht 33342 (blue): degenerated (A) and normal oocytes in germinal vesicle (GV - B), germinal vesicle break down (GVBD - C), and metaphase II (MII - D) state; oocyte staining with Mitotracker probe with a high (E) and low (F) fluorescence intensity (A-F: 50μm bar).

After IVM, oocytes were mechanically denuded and fixed in 1% glutaraldehyde for viability and chromatin configuration, and mitochondrial activity assays. Oocytes were stained by Hoechst 33342 (emission at 483 nm) and the oocyte viability and chromatin configuration were assessed by fluorescence microscopy (Eclipse 80 i, Nikon, Tokyo, Japan). The oocytes were classified as degenerated (DEG), germinal vesicle (GV), germinal vesicle breakdown (GVBD), and metaphase II (MII – Luz et al., 2013; Figure 2AD).

To evaluate the mitochondrial activity after IVM, the oocytes were incubated for 30 minutes in MitoTracker (0.5 μM) Orange CMTMRos (M7510, 38.5 °C and 5% CO2). Then, oocytes were mechanically denuded and fixed in 1% glutaraldehyde and were assessed by LM710 confocal microscope (Zeiss, Germany). The mitochondrial activity in each oocyte was evaluated by the fluorescence intensity, using Zen lite 2.3 SP1 software (Brevini et al., 2005; Figure 22F).

### Levels of progesterone

The spent media after maturation were collected and stored at -80 °C for progesterone assay. The concentrations of progesterone were measured from standard aliquots (200 µL), using the enzyme-linked fluorescent assay (ELFA), according to the manufacturer’s instructions (VIDAS® Progesterone, ref 30409).

### Statistical analyses

All statistical analyses were carried out using Sigma Plot version 11.0 (Systat Software Inc., USA). Data were reported as mean (±SEM) and percentage, and the results were considered significant when P<0.05. Comparisons of means were performed by Kruskal-Wallis test, whereas variables expressed as percentages were analyzed by chi-square or Fisher´s exact tests.

## Results

A total of 453 oocytes were distributed in three treatments: control, ethanol and *Folliculinum 6 cH*. The addition of ethanol (the vehicle of the homeopathic preparation) to the control IVM medium did not affect the percentage of degenerate, GV and GVBD and MII oocytes after IVM (Table 1). Moreover, no statistical difference was observed in the progesterone production among the treatments after IVM. In contrast, when compared to the control, *Folliculinum 6 cH* addition increased oocyte degeneration and reduced meiotic resumption rates (P < 0.05). Interestingly, the percentages of meiotic resumption and MII-oocytes were lower in the *Folliculinum 6 cH* treatment compared to its vehicle (ethanol treatment) (P < 0.05 - Table 1). Finally, when the treatments were compared, higher mitochondrial activity was observed in the ethanol treatment (P < 0.05 - Table 1).

**Table 1:** Chromatin configuration, mitochondrial activity and progesterone production (mean ± SEM) of sheep COCs after 24h of *in vitro* maturation in the absence (control) or presence of *Folliculinum 6 cH* or ethanol (homeopathic vehicle).

**Treatments**	**% DEG**	**%GV**	**%GVBD**	**%MII**	**Mitotracker (mean ± SEM)**	**Progesterone (ng/ mL)**
Control	9.6 (17/178)**^A^**	2.8 (5/178)**^A^**	87.6 (156/178)**^A^**	48.9 (87/178)**^AB^**	5.2 ± 1.4 ^B^	1.37**^A^**
*Folliculinum 6 cH*	21.9 (37/169)**^B^**	5.3 (9/169)**^A^**	72.8 (123/169)**^B^**	43.2 (73/169)**^A^**	7.4 ± 0.5 ^B^	1.58**^A^**
Ethanol	13.2 (14/106)**^AB^**	1.9 (2/106)**^A^**	84.9 (90/106)**^A^**	56.6 (60/106)**^B^**	11.8 ± 0.8 ^A^	2.68**^A^**

Different letters denote significant differences among treatment groups (P < 0.05). Degenerate (DEG), germinal vesicle (GV), germinal vesicle breakdown (GVBD) and metaphase II (MII). SEM: Standard Error of the Mean.

## Discussion

This study shows for the first time the effect of a homeopathic medicine, *Folliculinum 6 cH,* on oocyte *in vitro* maturation. The results clearly showed that for some endpoints *Folliculinum 6 cH* did differ from the controls including its vehicle (ethanol treatment).

*Folliculinum 6 cH* addition increased oocyte degeneration and reduced meiotic resumption (P < 0.05) when compared to the control treatment. The substance used to prepare *Folliculinum 6 cH* is the estrone, which is an estradiol precursor (Demarque et al., 2009). Some studies reported that *Folliculinum 6 cH* treats hormonal imbalance by stimulating estradiol secretion (Demarque et al., 2009; Legros, 2010). However, the results of the present study suggest that *Folliculinum 6 cH* supplementation to the control maturation medium might stimulate estradiol production, which in turn overstimulates the oocyte resulting in high rates of degeneration. However, the mechanism of action of homeopathic medicines, like *Folliculinum 6 cH*, remains to be elucidated.

Considering that, homeopathy is still a controversial topic, in the present study care was taken to avoid researcher bias by using a double-blind approach along with an *in vitro* culture technique (oocyte *in vitro* maturation). The *in vitro* maturation technique is an outstanding tool that allows objective analysis of oocyte chromatin configuration. In the present study, the efficiency of *Folliculinum 6 cH* was evaluated in the *in vitro* maturation using two controls, i.e., maturation medium (TCM199) and ethanol, which was the vehicle used for the preparation of *Folliculinum 6 cH*. Surprisingly, *Folliculinum 6 cH* treatment yielded lower mature oocyte rates compared to its vehicle (ethanol treatment). In agreement with previous results from our team (Lima et al., 2016), these results clearly showed that the effect of *Folliculinum 6 cH* was not due to its vehicle suggesting a different mechanism of action (Rughinis et al., 2018). It is well known that medium supplements, including hormones, growth factors, and antioxidants affect the efficiency of the *in vitro* culture of oocytes (Li et al., 2016; Veshkini et al., 2018) and embryos (Marques et al., 2010; Ashkar et al., 2010; Thongkittidilok et al., 2015) in a concentration-dependent manner. Even though the dynamization of folliculinum, i.e. 6cH used in the present study was not suitable for oocyte meiotic resumption, studies aiming to find out optimal concentrations of this component as well as other homeopathic products would be of great importance. Therefore, the use of homeopathy medicine in *in vitro* procedures, such as IVM, could be an alternative to improve oocyte maturation in many species. Moreover, the advantages of using homeopathic substances are due to the much lower cost and low toxicity of homeopathic medicines.

In the ethanol treatment, the oocytes presented the highest mitochondrial activity. The ethanol increases the concentration of cytoplasmic calcium ions (Ca^+2^ – Liu et al., 1998) and Ca^+2^ inside the cell acts as a second messenger, regulating important cellular events. Moreover, a single calcium increase can induce early oocyte activation events, such as resumption of meiosis and MII arrest, but not late events, such as pronuclear formation, and cleavage (Liu et al., 1998). Then, we suggest that increase of cytoplasmic Ca^+2^ concentrations by the ethanol, may increase the mitochondrial activity and the ATP production (Brookes et al., 2004), promoting meiosis resumption (Yu et al., 2010).

## Conclusion

In conclusion, *Folliculinum 6 cH* promoted oocyte degeneration and affect negatively the mitochondrial distribution, impairing meiosis resumption. Taken together, these results suggest that, at least for metaphase II rate and mitochondrial activity, the mechanism of action of *Folliculinum 6 cH* differs from its vehicle. Thus, this study opens new perspectives for the use of other homeopathic substances in *in vitro* maturation protocols.
